# Controllable Liquid Metal Microparticles Production and Patterning by Miniaturized Filter‐Sieve Generators

**DOI:** 10.1002/smtd.202500301

**Published:** 2025-06-02

**Authors:** Qingtian Zhang, Hongda Lu, Yipu Guo, Xiangbo Zhou, Liping Gong, Zexin Chen, Jialu Wang, Haiping Du, Shi‐Yang Tang, Weihua Li

**Affiliations:** ^1^ Faculty of Engineering and Information Sciences School of Mechanical Materials Mechatronic and Biomedical Engineering University of Wollongong Wollongong NSW 2522 Australia; ^2^ School of Electronic Computer and Telecommunications Engineering University of Wollongong Wollongong NSW 2522 Australia; ^3^ School of Mechanical and Manufacturing Engineering The University of New South Wales Sydney NSW 2052 Australia

**Keywords:** compact platforms, controllable sizes, high energy efficiency, high productivities, liquid metal microparticles

## Abstract

Liquid metal microparticles (LMMPs) with excellent conductivity and reactivity hold significant promise for applications in flexible electronics and sensors. However, current LMMP production methods face critical challenges, including achieving smaller particle sizes with low energy consumption, streamlining processes, and enhancing productivity. Herein, leveraging the tunable surface tension of LM droplets, a compact platform called the miniaturized filter‐sieve generator (MFSG) is presented, for scalable, energy‐efficient, and controllable LMMP production. The MFSG demonstrates high energy efficiency (average power consumption of 0.21 W) and productivity (6.51 × 10^5^ particles per minute) while enabling precise control of microparticle sizes (2–300 µm). Furthermore, the MFSG can uniformly produce LMMPs with varied compositions. Harnessing these capabilities, a reconfigurable platform integrating the MFSG is developed to enable complex droplet patterning and an LMMP‐based humidity sensor with high sensitivity. This innovative platform for on‐demand LMMP production with low energy consumption will drive significant advancements in electronic devices and sensing systems.

## Graphical abstract

A miniaturized filter‐sieve generator is introduced as an innovative, compact platform for scalable production and patterning of liquid metal microparticles. By leveraging a simple electrochemical mechanism, the MFSG achieves high productivity, energy efficiency, and precise size control. Its versatility is demonstrated in LM droplet patterning and the development of a highly sensitive humidity sensor.

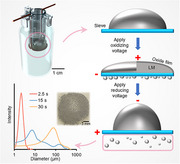

## 1 Introduction

Liquid metal microparticles (LMMPs), known for their exceptional electrical and thermal conductivities,^[^ ^1^ ^]^ hold great potential in diverse applications, including biomedicine,^[^ ^2^ ^]^ catalysis,^[^ ^3^ ^]^ actuators,^[^ ^4^ ^]^ and flexible sensors.^[^ ^5^ ^]^ The production of LMMPs with tailored properties is critical for expanding their applications and has drawn increasing attention. Controlling the size of LMMPs is crucial, as particle size directly influences electrical conductivity, mechanical flexibility, and integration precision. Smaller LMMPs allow for higher‐resolution patterning, improved mechanical compliance, and enhanced surface interactions, making precise size regulation essential for optimizing performance in advanced LM‐based technologies.^[^ ^6^ ^]^ Besides, a high‐productivity and straightforward fabrication process is expected to enhance cost efficiency, reproducibility, and practical implementation, making LM‐based technologies more accessible for large‐scale production.

Microfluidic platforms, as an on‐chip method, are widely employed to generate LMMPs within a size range of 50–200 µm due to their precise control over droplet size and monodispersity.^[^ ^7^ ^]^ To enhance precision, researchers have explored passive methods, such as modifying channel geometries and flow rates, and active approaches using external forces, including electrical, magnetic, acoustic, and piezoelectric fields.^[^ ^8^ ^]^ However, the high surface tension and oxide films of LM droplets pose significant challenges in microchannel systems, resulting in high‐pressure drops or channel blockages.^[^ ^9^ ^]^ Additionally, the fabrication of microfluidic chips involves complex photolithography processes.

To avoid the complicated fabrication, ultrasonication has emerged as a common top‐down, off‐chip method for LMMP production.^[^ ^10^ ^]^ This technique relies on disruptive forces to break large LM droplets into smaller particles, which are stabilized by the spontaneous formation of oxide skin on their surfaces.^[^ ^11^ ^]^ Ultrasonication offers simplicity and high‐power density, enabling efficient production of LM particles ranging from submicron to tens of microns in size. The particle size and stability can be tuned by adjusting parameters such as power, temperature, and duration.^[^ ^10c^ ^]^ Surfactants, such as polyethylene glycol (PEG), are often added to enhance stability and reduce particle size.^[^ ^12^ ^]^ However, the high sonication power (100–1000 W) leads to substantial energy loss, heat generation, environmental impact, and the formation of undesired gallium oxide nanorods.^[^ ^10a^ ^]^ Although centrifugation can reduce polydispersity after sonication, this additional step complicates the process and limits scalability.

To address these limitations, alternative methods have been developed. Shear forces generated by a spinning frustum or disk submerged in a carrier fluid have been used to fragment LM pumped through a needle.^[^ ^13^ ^]^ High voltages applied at the orifice overcome fluid surface tension, facilitating the production of LMMPs.^[^ ^13b^ ^]^ Reasonable adjustment of shear force, applied voltage, and feed rate of LM are key factors in controlling particle size. While this technique achieves high‐throughput production of uniform particles, it struggles to generate LMMPs smaller than 10 µm in diameter, even under elevated voltages.

Another promising approach leverages electrochemical or electrocapillary effects to fabricate LMMPs. This method allows rapid tuning of particle size by regulating the interfacial tension of LM streams via electrical potential.^[^ ^14^ ^]^ Under low voltages (e.g., 5 V), LM droplets exhibit reduced surface tension, enabling them to penetrate porous materials at room temperature.^[^ ^15^ ^]^ Utilizing this principle, researchers have developed high‐frequency LMMP generators capable of producing particles in the 40–200 µm range at a rate of 60 droplets per minute and an energy consumption of 2.77 × 10^−7^ kWh.^[^ ^14a^ ^]^ The particle size can be controlled by adjusting the applied voltage and replacing the exit orifice with different diameters. However, existing methods still face several challenges. Size control remains a key limitation, as most reported techniques struggle to reduce LMMPs below 10 µm while maintaining uniformity. Additionally, the low production rates and limited throughput make it difficult to integrate LMMPs into applications requiring high‐resolution patterning or flexible electronic systems. No existing works have explored the simultaneous use of alternating positive and negative voltages, which could provide greater dynamic control over LM droplet formation.

**Figure 1:**
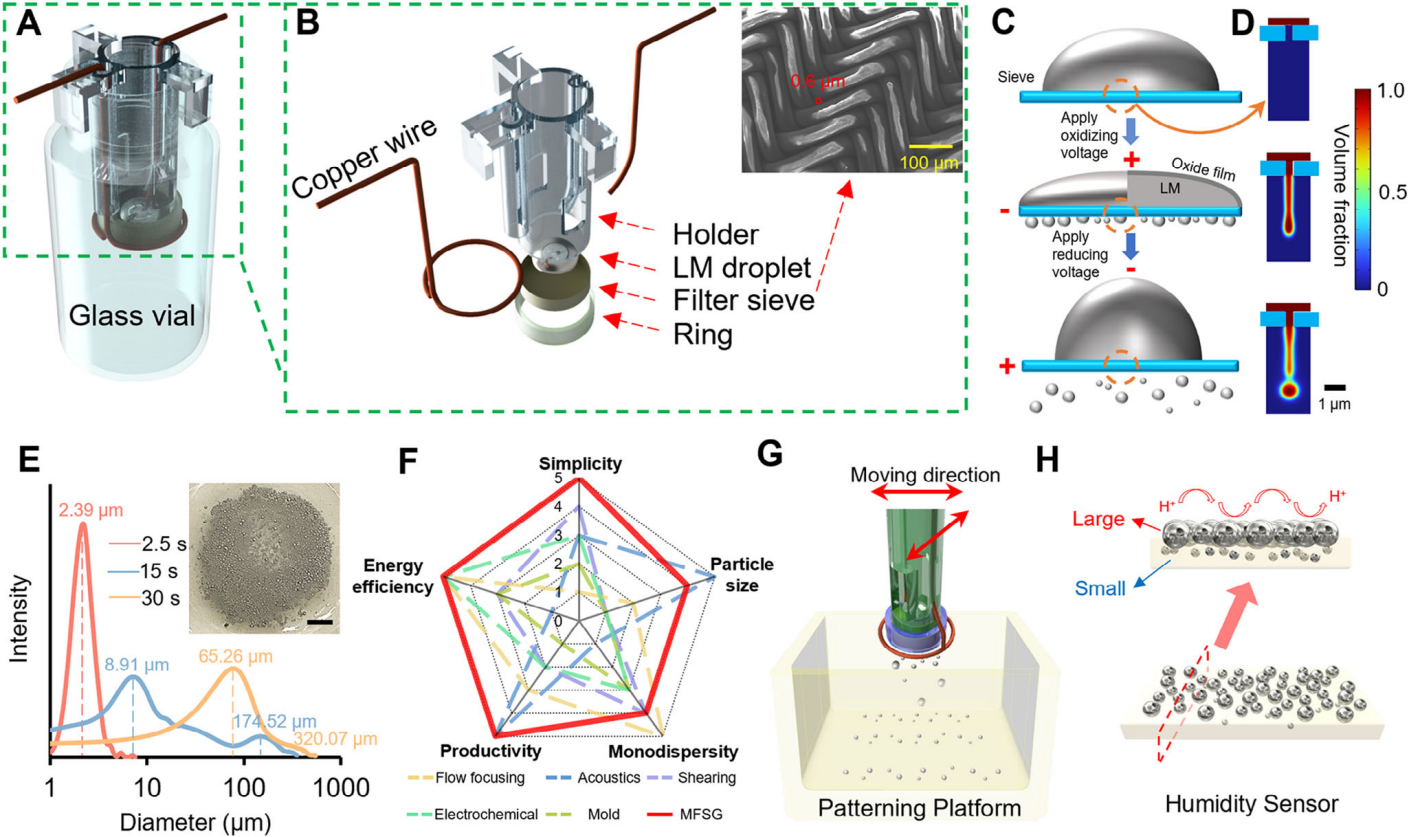
Design and performance of the MFSG. A) Schematic representation and B) exploded view of the MFSG, and the scanning electron microscopy (SEM) image of the sieve. C) Schematic representation of the working mechanism. D) Simulations for the LMMPs’ producing process. E) Size distributions of LMMPs produced by the MFSG under different oxidizing pulse periods and the optical image of the generated LMMPs. The scale bar is 3 mm. F) Comparison of the state‐of‐the‐art methods for fabricating LMMPs. The number on the axis represents the degree of each characteristic; “0” means lowest, while “5” means highest. The corresponding reference: flow focusing,^[^ ^9^ ^]^ acoustics,^[^ ^10a^ ^]^ shearing,^[^ ^13e^ ^]^ electrochemical,^[^ ^14b^ ^]^ molding.^[^ ^17^ ^]^ G) LMMPs pattern system based on the MFSG. H) A humidity sensor fabricated by LMMPs pattern system.

Here, we develop the miniaturized filter‐sieve generator (MFSG), a compact platform for the cost‐effective production of LMMPs with high productivity and tunable size distributions. By manipulating the surface oxide layer of LM droplets through an electrochemical mechanism,^[^ ^16^ ^]^ this method achieves precise control over particle size and distribution (**Figure** **1**A,B). Dynamically alternating oxidizing and reducing voltages facilitate LM droplets to pass through filter‐sieve apertures, forming uniform LMMPs (Figure 1C,D). The MFSG produces ultrafine LMMPs with a mean diameter of 2.39 ± 0.90 µm at an average power consumption of 0.21 W (2.06 × 10^−5^ kWh) and a high productivity rate of 6.51 × 10^5^ particles per minute. The size of LMMPs can be dynamically adjusted from 2.39 to 320.07 µm by modulating the oxidizing voltage duration (Figure 1E). Furthermore, the MFSG successfully fabricates LMMPs with diverse compositions, including Field's metal, Ga_50_In_50_, and pure Ga, demonstrating its versatility.

Compared to existing LMMP production techniques, the MFSG exhibits superior performance in producing fine, monodisperse particles with low energy consumption and high productivity (Figure 1F; Table S1, Supporting Information). We also developed a reconfigurable, automated platform integrated with the MFSG for on‐demand LM droplet patterning (Figure 1G). Additionally, the platform's potential is demonstrated through the development of a highly sensitive humidity sensor capable of accurately monitoring human respiration (Figure 1H). These advancements highlight the MFSG's potential to drive innovations in advanced sensors and manufacturing systems.

## 2 Results and Discussion

### 2.1 Development of the MFSG

A 3D schematic of the MFSG is shown in Figure 1A, with the corresponding optical image provided in Figure S1 (Supporting Information). The device comprises a commercial filter sieve with a hole size of 0.6 µm (Figure 1B), positioned at the bottom of a holder and secured by a fastening ring. An insulated nylon sieve is selected to avoid electrical field influence and electrochemical interference. A LM droplet is placed on the sieve. Two copper wires attached to the holder deliver alternating positive and negative voltages. The holder is immersed in a glass vial containing 1 m NaOH solution. To ensure complete contact between the LM droplet and the NaOH solution, two square openings are designed into the holder wall. The device's compact design eliminates the need for complex fabrication processes.

The working mechanism of the MFSG is illustrated in Figure 1C. Without an applied voltage, the LM droplet maintains an ellipsoidal shape on the sieve surface due to its high surface tension, as the oxide layer on the droplet is quickly removed by the NaOH solution.^[^ ^18^ ^]^ When an oxidizing (positive) voltage is applied, the surface tension of the LM droplet decreases significantly because of the interplay of electrochemical oxidation, surface‐active species formation and dissolution, and changes in interfacial charge distribution.^[^ ^15^, ^16^ ^]^ This reduction in surface tension allows the LM droplet to penetrate the sieve holes, forming pendent LM particles. Reversing the polarity of the voltage removes the oxide layer, rapidly increasing the surface tension and causing the pendent particles to detach from the sieve, forming LMMPs, as depicted in Figure S2 and Movie S1 (Supporting Information). The shape changes of an LM droplet under various applied voltages are recorded, suggesting the modulation of surface tension by electrochemical redox processes (Figure S3 and Text S1, Supporting Information).

To prevent the freshly formed LMMPs from merging in the NaOH solution after removing the oxide layer, 1 mL of PEG is added as a surfactant to stabilize the particles.^[^ ^19^ ^]^ Details regarding the LMMP generation process are provided in Figure S4a and Text S2 (Supporting Information). Simulations further confirm the feasibility of this mechanism, as shown in Figure 1D. Prolonged oxidizing treatment can also lead to LMMP formation through a dripping mechanism, where the growing mass of the pendent LM particle exceeds its surface tension limit. This phenomenon is discussed in Figure S4c and Text S2 (Supporting Information).

### 2.2 Performance Characterization of the MFSG

**Figure 2:**
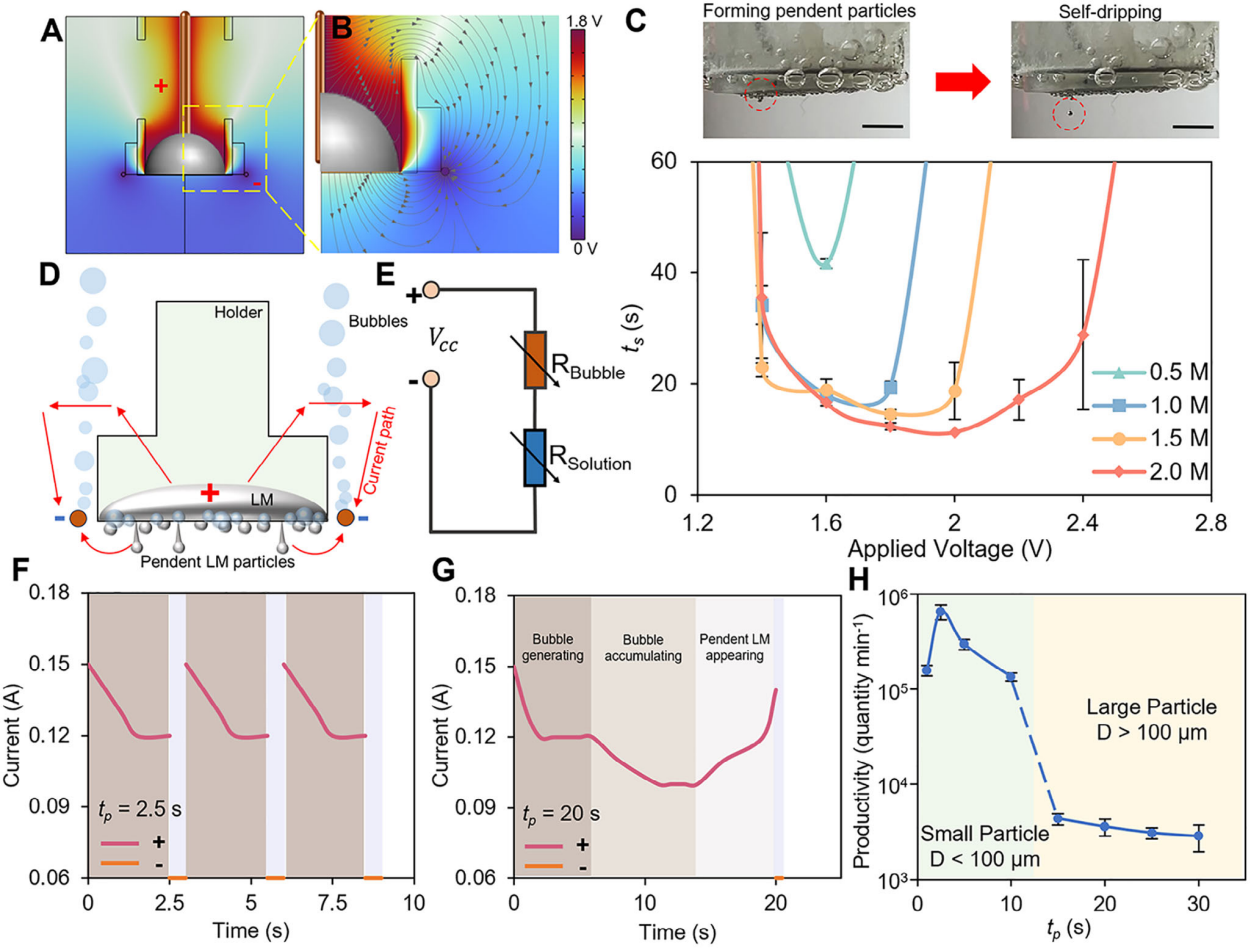
Performance characterization of the MFSG. Simulation of A) potential distribution and B) electric field distribution of the MFSG. C) Working range of the MFSG with different applied voltages and NaOH concentrations. The scale bars are 3 mm. The time that the droplets start to drip, denoted as “*t_s_*”, is recorded within 60 s. D) Schematic representation of the bubble generation and current paths while activating the MFSG. E) Equivalent circuit diagram of the device. *R_Bubble_* represents the resistance of the generated bubble “wall”. *R_Solution_* means the resistance of the electrolyte solution. Current‐time curves of the MFSG when the applying oxidizing pulse time (denoted as “*t_p_*”) is equal to F) 2.5 s and G) 20 s. H) Productivity of LMMPs using the MFSG with different *t_p_*.

After elucidating the working mechanism of the MFSG, we systematically explore and optimize its operational conditions. Simulations are conducted to evaluate the potential and electric field distributions across the MFSG when a voltage is applied to the copper wires (**Figure** **2**A,B). The insulated holder and securing ring notably impede electric field transmission. Current flows from the LM through the NaOH solution both upward and downward, ensuring adequate charge transfer. The upper path exhibits a stronger electric field than the lower path due to the sieve's presence.

We next investigate the effects of oxidizing voltage and NaOH concentration by measuring the initial dripping time (*t_s_*) of the LM droplet (Figure 2C). An increase in oxidizing voltage initially decreases *t_s_*, followed by an increase at higher voltages. This behavior is attributed to the formation of a bubble “wall” at elevated voltages, which hinders charge transfer (Figure 2D). Higher NaOH concentrations reduce *t_s_* by increasing charge density on the LM droplet's surface, thereby lowering its surface tension. The high‐concentration electrolyte also facilitates charge transfer, allowing the pendent LM particle to self‐drip even under high voltages. However, no dripping is observed within 60 s at oxidizing voltages below 1.4 V (the “dead zone”) due to insufficient charge transfer and potential difference. These findings indicate that moderate oxidizing voltages (≈1.8 V) and 1 m NaOH provide optimal conditions for stable LMMP production.

The influence of the negative electrode's position is also assessed. Simulations reveal that positioning the negative electrode 5 mm above the sieve results in the highest current density at the LM droplet–NaOH interface while positioning it 5 mm below the sieve results in the lowest (Figure S5, Supporting Information). A higher‐positioned negative electrode promotes greater charge transfer, forming a thicker oxide layer and reducing surface tension. As shown in Figure S6 (Supporting Information), placing the negative electrode 5 mm above the sieve achieves the fastest *t_s_* of 18.4 s, compared to 22.6 s when positioned below the sieve. Additionally, LM droplets tend to preferentially pass through sieve holes closer to the negative electrode, likely due to the electric field drawing the positively charged LM droplet toward the negative electrode.^[^ ^20^ ^]^

Energy efficiency is critical for ensuring the device's long‐term performance and reliability.^[^ ^21^ ^]^ We investigate the MFSG's energy consumption during LMMP production. The reduced voltage is set at 1.4 V with a 0.5 s duration to ensure complete LM droplet reduction during voltage switching.^[^ ^18^ ^]^ Current changes are recorded during operation (Figure 2F). Initially, the current decreases from 0.15 to 0.12 A due to the formation of a bubble “wall.” Over time, further bubble accumulation causes a decrease in current, followed by an increase as the growth of pendent LM particles reduces the gap between electrodes, lowering *R_solution_* (Figure 2G; Figure S7, Supporting Information). Larger applied voltages produce denser bubble walls, increasing *R_solution_*, as confirmed by equivalent circuit analysis (Figure 2E; Figure S8, Supporting Information). At an oxidizing voltage of 2.2 V, the current continuously decreases due to the dense bubble wall (Figure S9, Supporting Information). The average power consumption (*P_avg_*) is calculated as 0.2055 W and 0.1952 W for two working modes (*t_p_* = 2.5 s and 20 s, respectively; detailed in Text S3, Supporting Information).

The MFSG demonstrates high productivity, producing over 10^5^ LMMPs per minute for *t_p_* below 10 s (Figure 2H). Peak productivity of 6.51×10^5^ LMMPs per minute is achieved at *t_p_* = 2.5 s. However, at *t_p_* = 1 s, productivity drops to 1.58×10^5^ LMMPs per minute, likely due to insufficient time for the LM droplet to spread under oxidizing voltage. For *t_p_* >10 s, larger LMMPs (diameter >100 µm) are successfully collected at a rate exceeding 3000 particles per minute.

### 2.3 Versatile and Controllable LMMPs Production

**Figure 3:**
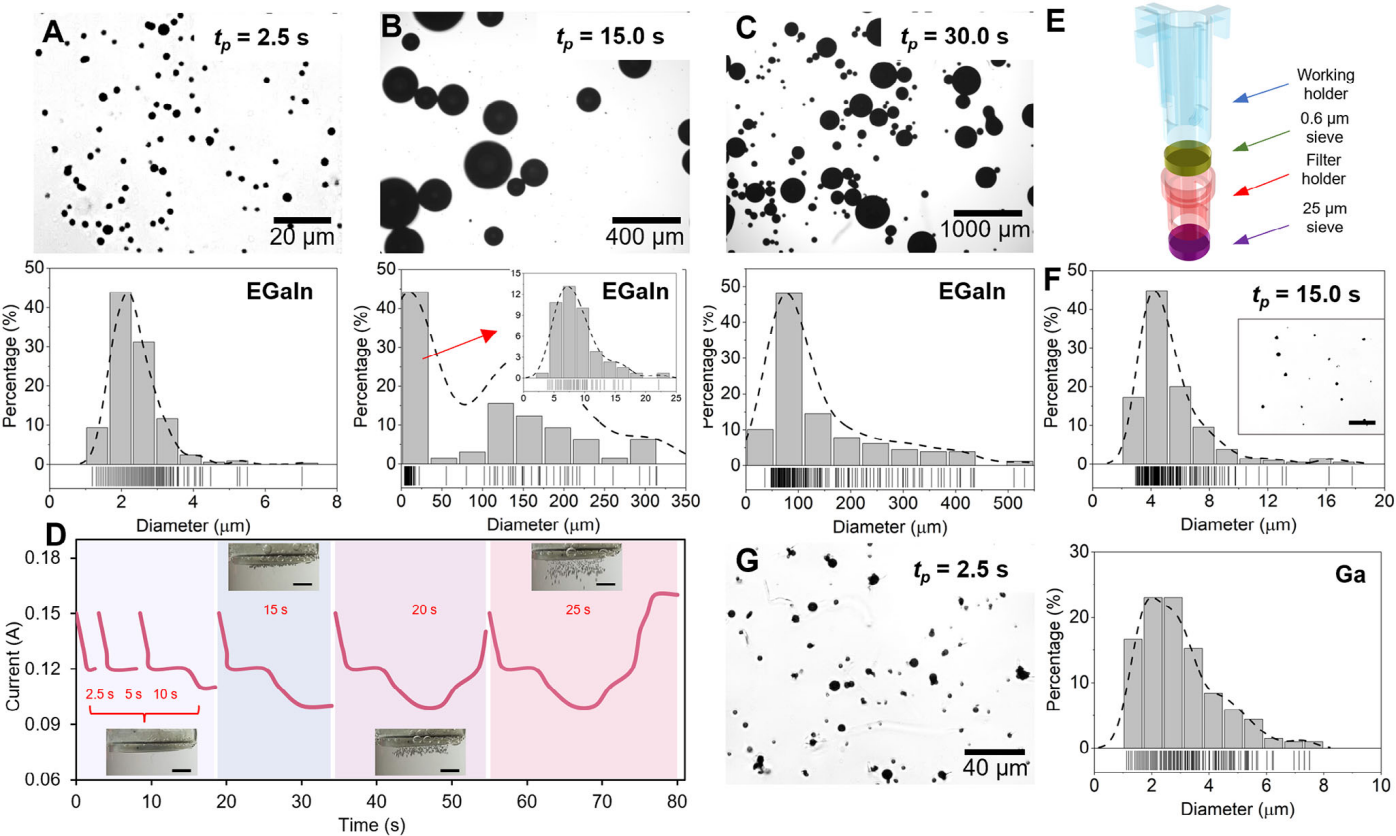
Production of LMMPs by MFSG. SEM images and size distribution of the LMMPs with a *t_p_* of A) 2.5 s, B) 15 s, and C) 30s. D) Current‐time curves of the MFSG when extending oxidizing pulse time *t_p_*. The scale bars are 3 mm. E) Schematic diagram of the MFSG with double sieve layers, and F) size distribution and SEM images of the produced LMMPs (*t_p_* = 15 s). G) Size distribution and SEM images of Ga MPs (*t_p_* = 2.5 s).

To achieve controllable LMMP production, we analyze the MFSG's performance at varying pulse durations. Optical microscope images and size distributions of LMMPs produced at different *t_p_* values are presented in **Figures** **3**A–C and S10 (Supporting Information). The results demonstrate that increasing *t_p_* produces larger LMMPs, as the droplets grow under gravity until truncation occurs (Text S2, Supporting Information). Table S2 (Supporting Information) summarizes the size and polydispersity index (PDI) of LMMPs for different *t_p_* values. Size control is achieved through a dynamic electrochemical approach, where alternating oxidizing and reducing voltages modulate the interfacial tension of the LM droplet, allowing it to deform and pass through the filter‐sieve apertures. By adjusting the oxidizing voltage period *t_p_*, we can precisely control the formation and detachment size of pendent LM particles, leading to reproducible and scalable microparticle generation (Movie S2 and Table S2, Supporting Information). At *t_p_* = 2.5 s, LMMPs achieve a minimum size of 2.39 µm with a highly monodisperse distribution (PDI = 0.14). As *t_p_* increases, polydispersity rises (Figure S10a,b, Supporting Information), with multiple peaks appearing in the size distribution at *t_p_* = 12.5, 15, and 17.5 s due to varying droplet velocities across the sieve (Figure 3B; Figure S10c,d, Supporting Information).

At *t_p_* = 20 s, some pendent particles overcome surface tension to self‐drip. This behavior merges the size distribution's three peaks into two, as larger LMMPs cease growing and stabilize. The average diameter of self‐dripping LMMPs is 312.64 µm (Figure S10e, Supporting Information). Extending *t_p_* to 30 s increases the proportion of smaller LMMPs compared to *t_p_* = 20 s, as secondary dripping occurs from previously formed positions. The average diameter of larger LMMPs stabilizes at 320.07 µm, comparable to *t_p_* = 20 s. Thus, longer *t_p_* results in a more dispersed size distribution, with the coexistence of large and small LMMPs.

The sieve's hole size also influences production. When comparing sieve diameters of 0.6 and 25 µm, similar LMMP sizes are observed at *t_p_* = 2.5 s. However, at *t_p_* >11 s, the larger hole size leads to significantly larger LMMPs, as pendent particles require more time to form (Figure S11, Supporting Information). We also provide a theoretical explanation for the close relationship between the size of the sieve hole and LMMPs (Text S4, Supporting Information).

We further explore dynamic LMMP production using the MFSG. Figure 3D and Movie S3 (Supporting Information) demonstrate smooth LMMP generation across *t_p_* values of 2.5, 5, 10, 15, 20, and 25 s, enabling size and concentration gradients. To enhance uniformity even under longer *t_p_*, we introduce a secondary sieve layer to reduce polydispersity and improve stability. A 3D‐printed column equipped with a 25 µm nylon sieve is added below the primary sieve to filter out LMMPs exceeding 25 µm in diameter (Figure 3E). This configuration produces LMMPs with an average diameter of 5.41 µm and productivity of 7708 particles per minute at *t_p_* = 15 s (Figure 3F). Unlike Figure 3B, the size distribution shows a single peak, as the second sieve effectively blocks larger LMMPs.

The MFSG's versatility extends to producing LMMPs with diverse compositions and melting points. For instance, pure Ga microparticles (MPs) with an average diameter of 2.96 µm are produced at a productivity of 2.38×10^5^ particles per minute (Figure 3G). These MPs are promising for biomedical applications, such as non‐invasive degradation of metallic implants,^[^ ^22^ ^]^ owing to Ga's ability to weaken grain boundaries in metals.^[^ ^23^ ^]^ Similarly, EGaIn LMMPs (average diameter: 2.59 µm) are produced at 2.87×10^5^ particles per minute using a 25 µm sieve (Figures S11 and S12, Supporting Information).

The MFSG also enables the production of Ga_50_In_50_ MPs (50 wt.% Ga and 50 wt.% In), a non‐Newtonian fluid with applications in flexible electronics and radiation shielding.^[^ ^24^ ^]^ Production is conducted at 70 °C using a water bath, yielding MPs with an average diameter of 6.88 µm and productivity of 3000 particles per minute (Figures S12 and S13, Supporting Information). Field's metal MPs are similarly produced at 70 °C, achieving a diameter of 5.67 µm and productivity of 4000 particles per minute (Figures S12 and S14, Supporting Information). The relatively lower productivity for Ga_50_In_50_ and Field's metal is attributed to their surface tension being less responsive to applied voltages.^[^ ^25^ ^]^

To verify the elemental composition and ensure material consistency, we conduct energy‐dispersive X‐ray spectroscopy (EDS) analysis on the produced LMMPs, including EGaIn, Ga, Ga_50_In_50_, and Field's Metal. The results confirm that LMMPs derived from EGaIn, Ga, and Field's Metal exhibit consistent elemental distributions matching their precursor compositions (Figure S15, Supporting Information). However, for Ga_50_In_50_ LMMPs, the EDS analysis reveals a higher proportion of Ga than the nominal 50:50 Ga/In ratio. This deviation could be attributed to the non‐Newtonian behavior of Ga_50_In_50_, which affects the flow dynamics and droplet formation during microparticle generation.^[^ ^24a^ ^]^ As a result, during electrochemical processing, the flowing and detaching portion of the LM becomes enriched in the eutectic gallium‐indium component, while the higher‐indium phase may remain within the bulk.

To demonstrate the durability of the MFSG under prolonged operation, we conducted a continuous 1‐h experiment and evaluated potential filter degradation, electrolyte dilution, and performance drift. Figure S16 (Supporting Information) shows the optical and SEM images of the filter sieve before and after the experiment, followed by a NaOH cleaning process to remove any accumulated residues. The result suggests that the structural integrity of the filter remained intact, with no significant deformation or clogging observed. To evaluate potential electrolyte dilution over extended operation, we measure the pH value of the NaOH solution before and after 1 h of continuous use. The results indicate that the pH remained stable, suggesting no significant dilution or depletion of the electrolyte (Figure S17, Supporting Information). This stability ensures consistent electrochemical conditions throughout prolonged operation. A video records the start of operation and after 1 h of continuous working to compare the MFSG performance (Movie S4, Supporting Information). The *t_p_* is set to 15 s per cycle. After 1 h of operation, additional EGaIn is introduced onto the filter sieve, and the device successfully continues to generate LMMPs without any noticeable degradation in performance. These results confirm that the MFSG maintains stable functionality over extended use.

### 2.4 Applications of the Produced LMMPs

**Figure 4:**
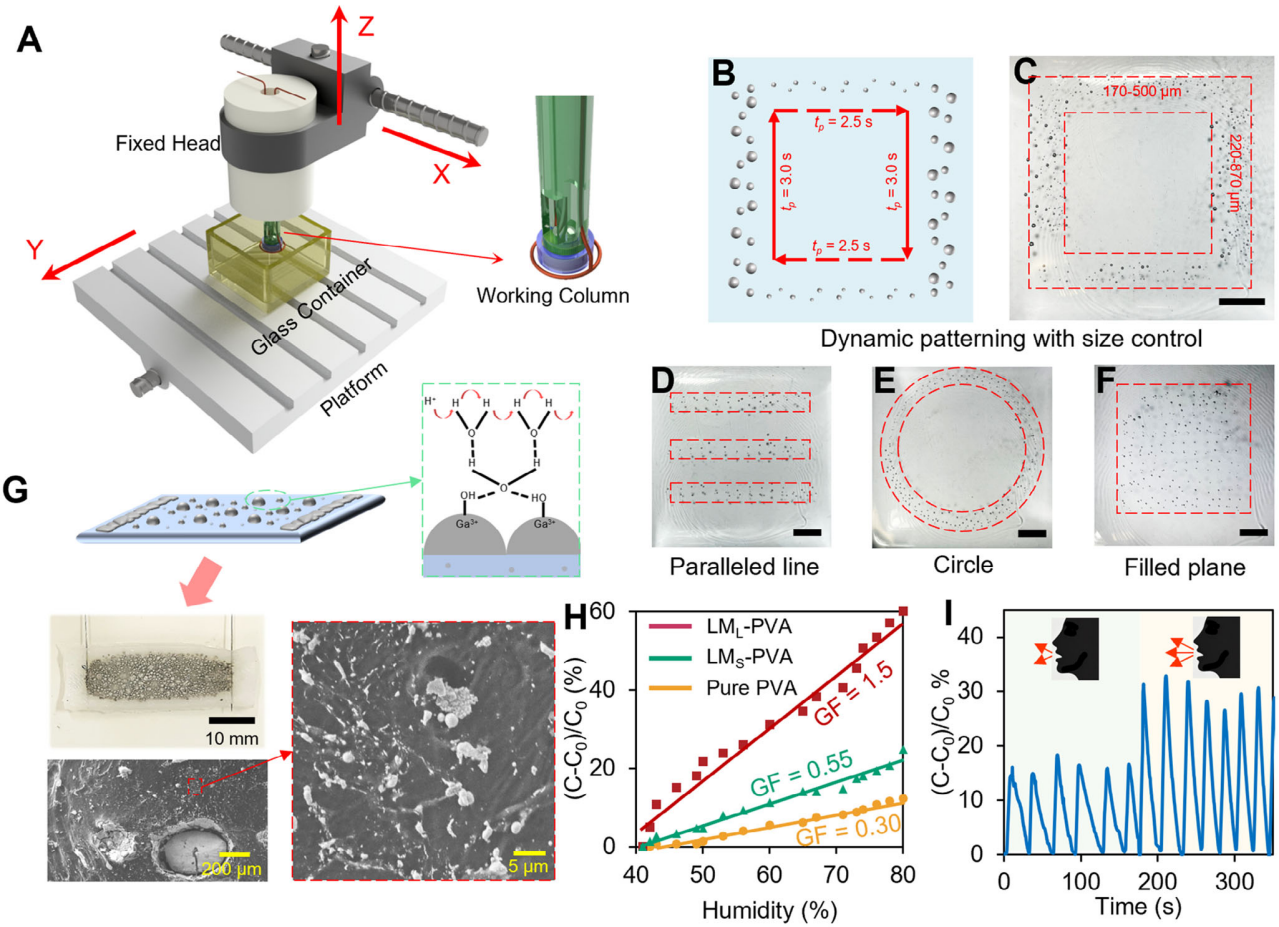
Development of an automatic platform for LMMPs patterning and LMMPs‐based humidity sensor. A) Schematic diagram of the platform. “X”, “Y”, and “Z” indicate the moving direction. B) Schematic and C) Optical image of LMMPs pattern of LM droplets. Programmable pattern with the different shapes of D) square, E) circle, and F) filled plane. The scale bars are 10 mm. G) Schematic and optical image of LM droplets‐based humidity sensor and SEM image of its cross‐section. H) Relative capacitance changes versus humidity of LM PVA hydrogels (LM_L_‐PVA is denoted as hydrogel with large LMMPs, and LM_S_‐PVA is denoted as hydrogel with small LMMPs) and pure PVA hydrogel. I) The dynamic capacitive response of the humidity sensor when performing a blow‐release test.

The patterning of LM is critical for enabling diverse applications in fields such as flexible electronics, soft robotics, biomedical devices, and thermal management systems.^[^ ^26^ ^]^ Leveraging the high performance of the MFSG, we develop a reconfigurable and automated platform for on‐demand LM droplet patterning (**Figure** **4**A). By dynamically controlling the *t_p_*, the platform generates LMMP patterns of varying sizes in a square configuration (Figure 4B,C). The feed speed (V_F_) of the mobile port can also be determined to control the space of the adjacent LM droplets, resulting in customized patterns of LM droplets (Figure S18, Supporting Information). The high packing density of the pattern is showcased in Figure S18 (Supporting Information) since the tiny LMMPs are generated simultaneously around the large LMMPs. Additionally, the platform demonstrates excellent controllability by producing LM droplet patterns in various shapes, including lines, squares, circles, “S” shapes, and full square planes (Figure 4D–F; Figure S19, Supporting Information). The MFSG's ability to produce fine LMMPs with high productivity offers the potential for large‐area precision patterning.

Using the automated platform, we also fabricate a humidity sensor incorporating LMMPs. The fabrication process involves spreading 2 mL of 10 wt.% polyvinyl alcohol (PVA) solution on the bottom of a 3D‐printed mold, followed by pouring 12 mL of 1 m NaOH solution into the mold. The high viscosity of the PVA solution ensures its stability beneath the NaOH solution. Once the platform is activated, LMMPs with desired sizes drop to the interface between the two solutions and settle through the PVA layer under gravity. As reported in our previous work, the size of LMMPs influences their distribution within the PVA hydrogel matrix.^[^ ^27^ ^]^

For large *t_p_* values, the MFSG generates LMMPs of various diameters, resulting in a gradient structure within the hydrogel (Figure 4G). Larger LMMPs (200–400 µm) accumulate at the bottom of the matrix, while smaller LMMPs (2–10 µm) are distributed throughout. This gradient structure enhances the hydrogel's surface for dynamic humidity detection. The sensing mechanism relies on electron transfer via the Grotthuss chain reaction.^[^ ^28^ ^]^ The Ga oxide surface absorbs water molecules through hydrogen bonding at high humidity levels,^[^ ^29^ ^]^ leading to an increased dielectric constant and, consequently, higher capacitance.^[^ ^30^ ^]^

Compared to pure PVA hydrogels, the inclusion of LMMPs significantly enhances sensitivity to humidity changes. This is quantified by the gauge factor (GF), defined as the ratio of relative capacitance change to relative humidity change, which is five times higher for LMMP‐filled PVA than for pure PVA hydrogels (Figure 4H). Notably, a hydrogel with small LMMPs also displays a low GF due to the low gravity of small LMMPs, leading to insufficient distribution for effective electron transfer. The developed LMMP‐based humidity sensor exhibits outstanding performance and ease of fabrication compared to existing humidity sensors (Table S3, Supporting Information).^[^ ^31^ ^]^ Harnessing its high sensitivity, the sensor is applied to detect human exhalation. The results demonstrate dynamic and reliable humidity detection, as shown in Figure 4I, confirming the sensor's stability and robustness.

## 3 Conclusion and Outlook

In this study, we develop an MFSG for the efficient, scalable production of uniform LMMPs. By alternating oxidizing and reducing voltages, the surface tension of the LM droplet is controlled, enabling it to diffuse through the sieve's hole to generate LMMPs, with controllable sizes by adjusting the oxidizing pulse duration (yielding diameters from 2.39 to 320.07 µm for *t_p_* values of 2.5–20 s).

We conduct a comprehensive investigation of the MFSG's working conditions, demonstrating the MSFG's capability to produce ultrafine LMMPs with diameters as small as 2.39 µm at high productivity (6.51 × 10^5^ particles per minute) and low energy consumption (average power of 0.21 W). Additionally, the MFSG showcases versatility by successfully fabricating LMMPs from diverse materials, including Field's metal, Ga_50_In_50_, and pure Ga. Moreover, an automated platform incorporating the MFSG is developed to enable customizable LM droplet patterns. Notably, the fidelity and density of the patterns are critical for integration into densely packed circuit structures and soft electronics.^[^ ^32^ ^]^ We believe this new patterning strategy offers a strong foundation, and it is possible to refine it by reducing sieve aperture size and designing tailored sieve architectures to achieve the desired densely packed microstructures, thereby meeting the increasing demands of next‐generation soft electronic applications. This platform is further utilized to fabricate a highly sensitive humidity sensor capable of accurately detecting human respiration.

While our current technique is optimized for microscale LM particle fabrication, it also holds the potential for generating sub‐micron or nanoscale particles through further refinement. One possible strategy is to improve the sieve manufacturing process, such as by using advanced microfabrication techniques to reduce aperture sizes. For instance, photolithography can theoretically achieve apertures as small as 100 nm, enabling the formation of nanoscale LM droplets.^[^ ^33^ ^]^ Alternatively, introducing surfactants during electrochemical synthesis may help stabilize interfacial dynamics and suppress droplet coalescence, contributing to the formation of smaller particles.^[^ ^10a^ ^]^ These directions, though technically challenging, represent promising avenues for expanding the versatility of the proposed system toward nanoscale LM particle generation.

We believe that this streamlined and innovative technique will significantly expand the potential applications of LMMPs. It will drive progress not only in patterned integration for flexible and stretchable electronics, but also in the preparation of LM‐based emulsions for emerging biomedical applications, such as drug delivery, cancer therapy, and bioimaging.^[^ ^2^, ^34^ ^]^ By combining high productivity, precise size control, and energy efficiency, the MFSG offers a transformative approach to LMMP production and paves the way for advancements across multiple disciplines.

## 4 Experimental Section

### Materials

Gallium and indium (99.99%) were purchased from EBAY, Australia. Polyvinyl alcohol (PVA, Mw 14 600–18 600, 99+%, hydrolyzed), solid NaOH, and Polyethylene glycol (PEG, average mol wt. 200) were purchased from Sigma–Aldrich, USA. Nylon filter sieves (hole sizes: 0.6 and 25 µm) were purchased online.

### Experimental Equipment and Tools

Two 0 to 30 V DC‐regulated power supplies, two Arduino‐compatible 5 V relays, and an Arduino UNO microcontroller (Jaycar, Australia) were used to periodically apply oxidizing voltage and reduction voltage on the LM sieve. An OLYMPUS CKX41 optical microscope was used to observe the LMMPs, and an optiMOS camera was used to take the images of them. SEM images of LMMPs and nylon sieves were taken by JEOL JSM‐6490LA. An S.E.M Magnetic Stirrer Hot Plate was used to heat the LM sieve to melt the Field's metal and Ga_50_In_50_. The videos of the LMMPs generation were recorded by Chronos 1.4 High‐Speed camera. The numerical simulations were performed by the COMSOL 5.2 software package. A VG‐S3018 CNC engraving machine and Candle software were used to control the pattern of LM droplets.

### Fabrication of MFSG

EGaIn was fabricated by mixing gallium (75.5 wt.%) and indium (24.5 wt.%). Solid NaOH was dissolved in deionized water to form a 1 m solution. The working holder was fabricated by a 3D printer (Formlabs Form 3), and the material was resin (Clear V4). The holder and securing ring were ultrasonically treated in ethanol at room temperature for 10 min before use to avoid residual resin. The applied voltage was controlled by two independent power supplies, two electromagnetic relays, and a microcontroller (Figure S21, Supporting Information). Two copper wires (diameter of 0.53 mm, length of 200 mm) were used as the positive and negative electrodes of the generator. One copper wire with a looped end was mounted on the bottom of the holder, while the other one was directly inserted in the holder to touch the LM droplet. A 15 × 15 mm nylon sieve was fixed on the holder by a securing ring. 250 µL EGaIn was added into the working holder, and then a copper wire was inserted into the EGaIn droplet, while another copper wire was bent to fit snugly on the ring and be in the same plane as the nylon sieve. 1 mL PEG was poured into the 20 mL glass vial to stabilize the produced LMMPs, and then 18 mL NaOH solution was slowly added. The assembled working holder was finally put in the vial.

### Generation of LMMPs by MFSG

For producing LMMPs, moderate oxidizing voltages (1.8 V) and NaOH solution (1 m) was used. The reducing voltage was set to 1.4 V with a duration of 0.5 s to remove the oxide layer of LM. The oxidizing power time *t_p_* varied from 1 to 30 s. 1 mL PEG was added as the surfactant to stabilize the LMMPs. The EGaIn MPs were produced with the fine sieve (0.6 µm) at room temperature, while Ga, Ga_50_In_50_, and Field's metal MPs were produced with the coarse sieve (25 µm) at 70 °C.

### Patterning of LMMPs

The LMMPs patterning platform used the sieve with hole sizes of 25 µm and applied an oxidizing voltage of 2.0 V for 2.5 or 3 s and a reducing voltage of 1.4 V for 0.5 s. The holder was assembled on the mobile port of the XYZ motorized stage. A square glass container (bottom size of 65 × 65 mm) filled with 1 m NaOH solution was placed on the mobile plate as the large pattern area. To increase the resolution of the LM droplet pattern, the working holder was replaced with a thinner inner diameter (5 mm). The distance among adjacent LM droplets could be adjusted by the feed speed (V_F_) of the mobile port to realize customized patterns. The V_F_ in Figure 4C and Figure S18 (Supporting Information) was 1.33 mm s^−1^, while in Figure 4D–F and Figure S19 (Supporting Information) was 1 mm s^−1^.

### Fabrication of Humidity Sensor

In the LMMPs patterning platform, 2 mL 10 wt.% polyvinyl alcohol (PVA) solution was spread on the bottom of a 3D printed mold with a length of 40 mm, width of 15 mm, and height of 30 mm, and then 12 mL 1 m NaOH solution was added into the mold. An oxidizing voltage of 2.0 V and a reducing voltage of 1.4 V were applied. The LM‐PVA hydrogel containing larger LMMPs was fabricated with a *t_p_* of 15 s, whereas the hydrogel with smaller LMMPs was fabricated with a *t_p_* of 2.5 s. The V_F_ was set as 0.5 mm s^−1^ to ensure the dense arrangement of LMMPs. After patterning the LMMPs, the NaOH solution in the mold was removed, and the remaining solution containing PVA and LMMPs was placed in a 70 °C oven and heated for 6 h. The obtained xerogel was soaked in deionized water for 24 h to reach equilibrium.

## Conflict of Interest

The authors declare no conflict of interest.

## Supporting information



Supporting Information

Supplemental Movie 1

Supplemental Movie 2

Supplemental Movie 3

Supplemental Movie 4

## Data Availability

The data that support the findings of this study are available from the corresponding author upon reasonable request.
