# Supplementary material for: Controllable Liquid Metal Microparticles Production and Patterning by Miniaturized Filter‐Sieve Generators
Source: Small Methods. 2025 Jun 2;9(11):2500301. doi: 10.1002/smtd.202500301 (PMC12641367; doi:10.1002/smtd.202500301)
Supplement: Supplementary file 1 — Supporting Information [file SMTD-9-2500301-s005.docx]

Supporting Information

**Controllable Liquid Metal Microparticles Production and Patterning by Miniaturized Filter-Sieve Generators**

Qingtian Zhang, Hongda Lu*, Yipu Guo, Xiangbo Zhou, Liping Gong, Zexin Chen, Jialu Wang, Haiping Du, Shi-Yang Tang*, and Weihua Li*

Q. Zhang, Dr. H. Lu, Y. Guo, X. Zhou, L. Gong, Z. Chen, J. Wang, Prof. W. Li

Faculty of Engineering and Information Sciences

School of Mechanical, Materials, Mechatronic and Biomedical Engineering

University of Wollongong

Wollongong, NSW 2522, Australia

Prof. H. Du

School of Electronic, Computer and Telecommunications Engineering

University of Wollongong

Wollongong, NSW 2522, Australia.

Dr. S.-Y Tang

School of Mechanical and Manufacturing Engineering

The University of New South Wales

Sydney, NSW 2052, Australia

*Corresponding authors. E-mail: weihuali@uow.edu.au (W. Li), hongdal@uow.edu.au (H. Lu), shiyang.tang2@unsw.edu.au (S. Tang).

**Supplementary Text**

Supplementary text 1. Effect of electrochemical oxidation-reduction on LM droplet shape.

To explain how oxidation modulates surface tension, we utilize the concept of capillary length, which represents the ratio between gravitational and interfacial forces.[17] The capillary length can be calculated by

$$\begin{aligned} L=\sqrt{\frac{\gamma}{\rho g}}\#\left( 1 \right) \end{aligned}$$

where the $L$ is characteristic capillary length, $\gamma$ is the surface tension, $\rho$ is the density of the EGaIn, and $g$ is the acceleration due to gravity. The characteristic radius (*R*) of a droplet is defined as the observed radius of the LM droplet under different experimental conditions. When $L<R$, gravity dominates, leading to a flattened droplet shape. When $L>R$, surface tension dominates, maintaining a more spherical droplet. Our experimental results show that oxidation decreases surface tension, leading to a lower capillary length and increased droplet spreading (Figure S3). Conversely, in NaOH, where oxide removal occurs, the higher surface tension results in an increased capillary length and more pronounced shape retention of the LM droplet.

Supplementary text 2. Force analysis of pendent LM particles.

Intrinsically, surface tension, gravity, buoyant, and viscous drag are the main forces determining motion of LM droplet. In this study, the viscous drag between LM and sieve and buoyant of LM droplet is neglected. The schematic diagram of force analysis is shown in Figure S4. There are three situations of the generation of the LMMPs: (1) the pendent LM particle is pulled apart by the sudden increase in surface tension (Figure S4a); (2) pendent LM particle in peripheral position is pulled apart to drop (Figure S4b); (3) pendent LM particle self-drops due to the increase in weight (Figure S4c). In the first scene, after LM droplet is applied oxidized voltage and the oxidizing power time is $t_{1}$, the force equation can be expressed as

$$\begin{aligned} F_{\gamma} = m_{LM}\left( t_{1} \right)g+m_{LM}\left( t_{1} \right)a\#\left( 2 \right) \end{aligned}$$

where $F_{\gamma}$ is the force induced by LM interfacial tension, $m_{LM}(t_{1})$ is the mass of the LM droplet at the moment of $t_{1}$, $a$ is the acceleration of the LM droplet. $F_{\gamma}$ can be calculated by

$\begin{aligned} F_{\gamma}=\pi d\gamma sin\theta\#\left( 3 \right) \end{aligned}$where $d$ is the diameter of the smallest circle of the pendent LM particle neck, $\gamma$ is the surface tension of LM, $\theta$ is the contact angle between the pendent LM particle neck and NaOH solution. When the LM droplet is applied reduced voltage at $t_{1}$, the $\gamma$ is greatly increased to $\gamma'$, which the increase was recorded from ~0 mN m^-1^ to ~500 mN m^-1^ (*25*). The volume of the pendent LM particle is not further increase while an instantaneous acceleration $a'$ appears. So the equation can be displayed as

$$\begin{aligned} F_{\gamma}'=\pi d\gamma'sin\theta= m_{LM}\left( t_{1} \right)g+m_{LM}\left( t_{1} \right)a'\#\left( 4 \right) \end{aligned}$$

Thus, the pendent LM particle can overcome the tension limit of the slender pendent LM particle neck and drop as the LMMP. It is worth to note that the pendent LM particles in peripheral position seem to have been pulled apart to drop (Figure S4b). When the oxidized LM droplet is applied with reducing voltage, it morphologically changes from flat form to approximate sphere in a short time. Therefore, the pendent LM particles in the reduced part of the LM droplet bottom surface are broken due to a sudden increase in surface tension. In addition, if the oxidizing voltage applies to the LM droplet without applying reduction voltage, the volume of the LM particle would continuously enlarge (Figure S4c). We assume that $t_{2}$ is the certain moment just before the pendent LM particle drops. In this case, the force equation of can be express by

$$\begin{aligned} F_{\gamma} = m_{LM}\left( t_{2} \right)g+m_{LM}\left( t_{2} \right)a\#\left( 5 \right) \end{aligned}$$

After that moment, the downward force of the pendent LM particle exceeds the tension limit of pendent LM particle neck, resulting in the drop of the LMMP. In conclusion, the size of the LMMP can be controlled by the duration of applying the positive voltage (*t_p_*). The larger *t_p_* is, the larger the size of LMMPs is. The size of LMMP reaches its maximum when *t_p_* is increased to the point where the gravity is large enough to overcome the tension of LM droplet. After this, the LMMP falls, and another one regenerates.

Supplementary text 3. The average power of MFSG.

The $P_{avg}$ over a time interval can be calculated by integrating the power over the time interval and then dividing by the total duration of the time interval. Mathematically, this can be expressed as

$$\begin{aligned} P_{avg}= \frac{1}{T}\int_{0}^{T} P\left( t \right)dt\#\left( 6 \right) \end{aligned}$$

where 𝑃(𝑡) is the power at time 𝑡 and 𝑇 is the total time duration. For discrete data points, a method when dealing with time-series data is to use the trapezoidal rule for numerical integration:

$$\begin{aligned} P_{avg}= \frac{1}{T}\int_{0}^{T} P\left( t \right)dt= \frac{1}{T}\sum_{i=0}^{n-1} \frac{P_{i}+ P_{i+1}}{2}\Delta t_{i}\#\left( 7 \right) \end{aligned}$$

where $P_{i}$ and $P_{i+1}$ are the power values at consecutive time points, and $\Delta t_{i}$ is the time interval between these points.

Supplementary text 4. The relationship between size of sieve hole and size of LMMP.

When applied oxidizing voltage on the LM droplet, its surface tension dramatically decreases to ~0 mN m^-1^. It begins to flow through the hole on the sieve and a pendent particle form when its gravitational force becomes comparable to the surface tension force. Simplifying the calculation, we hypothesize that LM droplet is spherical. The force equation can be expressed as

$$\begin{aligned} \rho_{LM}g\cdot\frac{4}{3}\pi R^{3}=2\pi r\gamma+\rho_{s}g\cdot\frac{4}{3}\pi R^{3}\#\left( 8 \right) \end{aligned}$$

Where $\rho_{LM}$ is density of LM, $\rho_{s}$ is density of the solution, $R$ is the radius of the LM pendent particle, $r$ is the radius of the sieve hole, and $\gamma$ is the surface tension. Therefore,

$$\begin{aligned} R=\sqrt[3]{\frac{3\gamma}{2(\rho_{LM}-\rho_{s})g}\cdot r}\#\left( 9 \right) \end{aligned}$$

At this point, the application of a reducing voltage facilitates droplet detachment. Revealing reducing the size of sieve hole holds the possibility to achieve production of nanoscale LM particles.

**
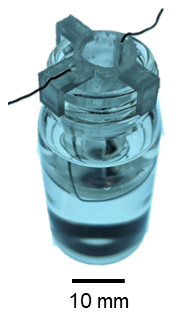
**

**Figure S1.** The actual image of the MFSG.


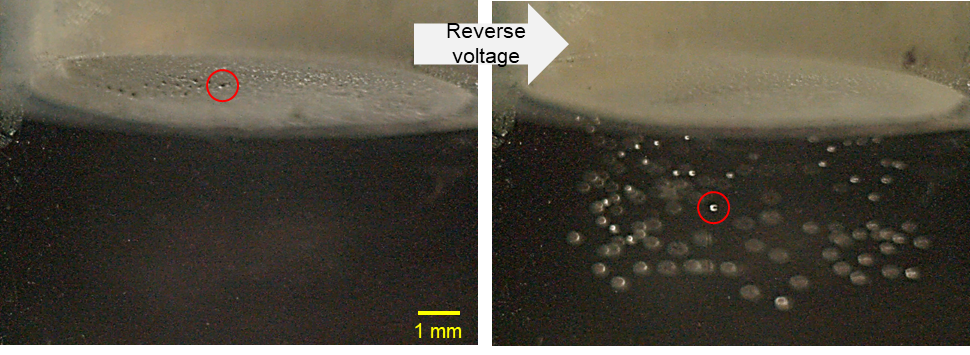


**Figure S2.** The photography of LMMPs generated.


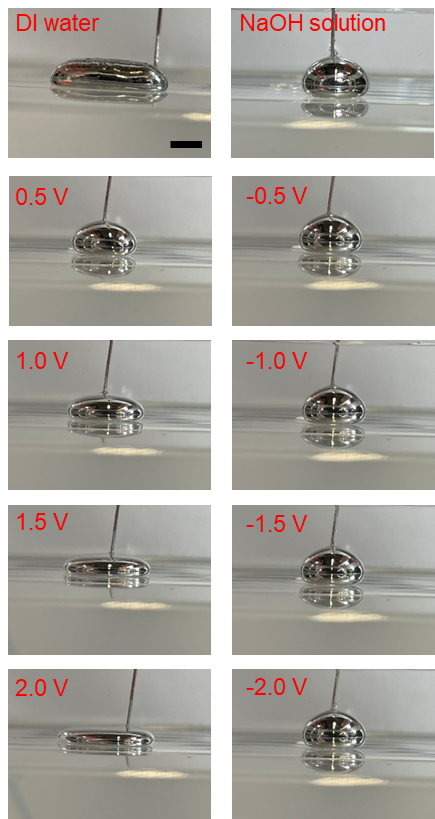


**Figure S3.** The optical images of the 200 µL LM droplet in DI water, NaOH solution, and under different voltages. The LM droplet is placed on an acrylic box and connected with a copper wire. The scale bar is 5 mm.


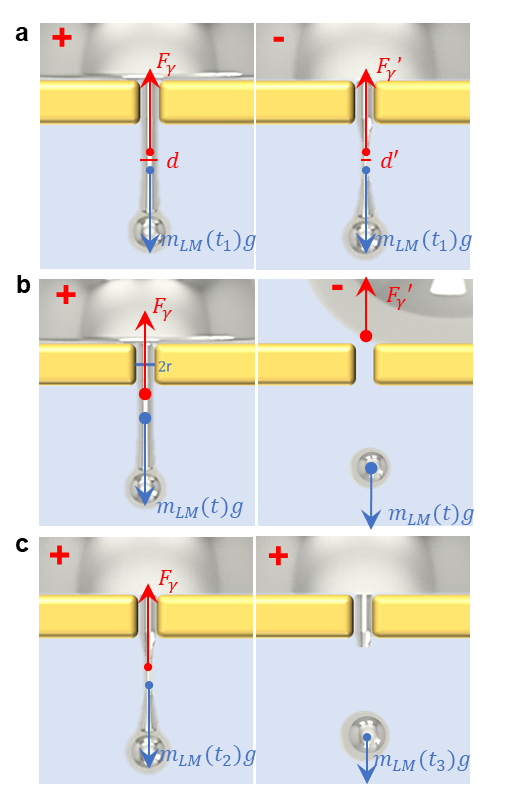


**Figure S4.** Force analysis of LM particles while the device is working. (a) pendent LM droplet dropped by reduction voltage. (b) pendent LM droplet in peripheral position is pulled apart to drop. (c) pendent LM droplet begin to self-drop.


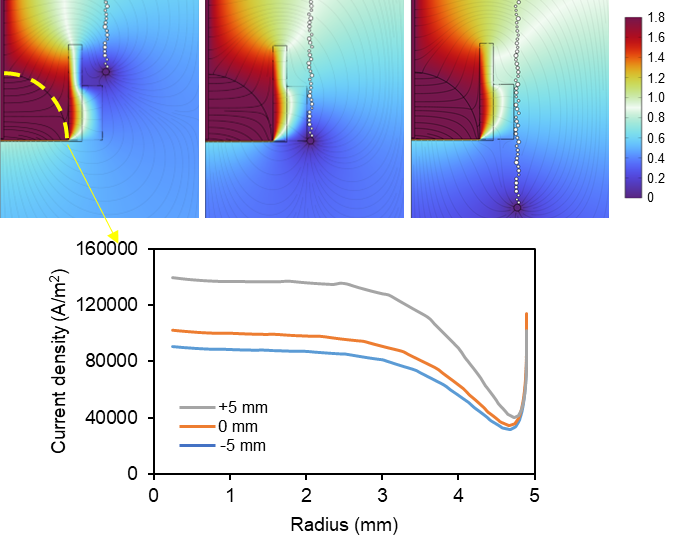


**Figure S5.** Numerical simulations about current density distribution on the upper interface of LM droplet and NaOH solution when negative electrode placed in different position.

**
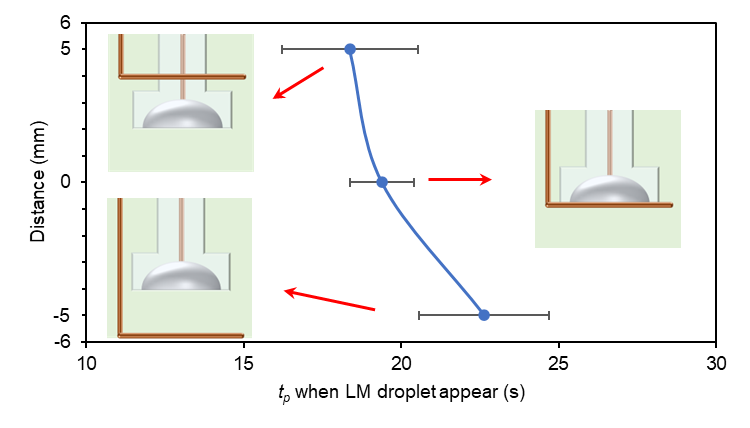
**

**Figure S6.** The effects of the position of the negative electrode to self-dropping time of pendent LM particles.

**
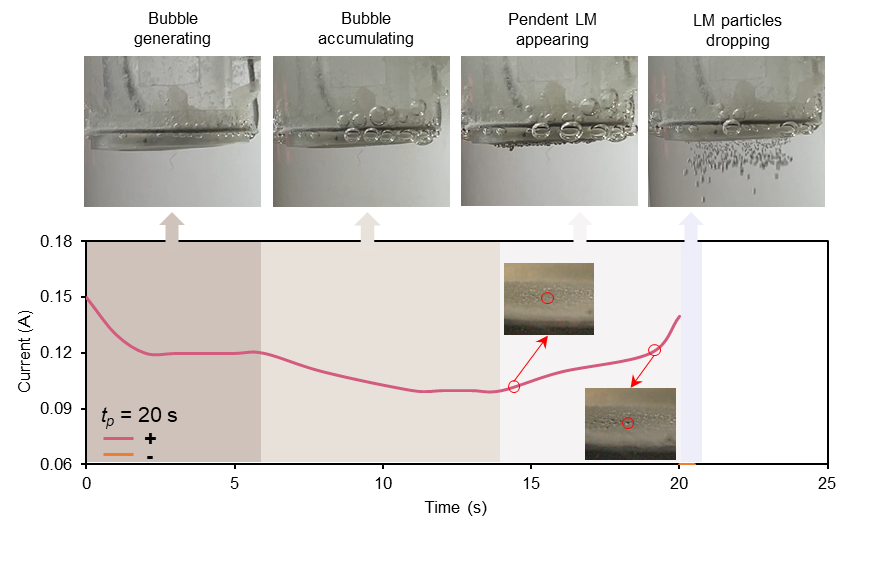
**

**Figure S7.** The electrical behavior during MFSG working. First, bubble generation increases the equivalent circuit resistance, leading to a decrease in current. As bubbles accumulate, the current continues to drop. Then, the formation of a pendant LM droplet reduces the distance between the anode and cathode, lowering the resistance and causing the current to increase. When the LM particles detach, the distance between the anode and cathode rapidly increases, resulting in a sharp drop in current.


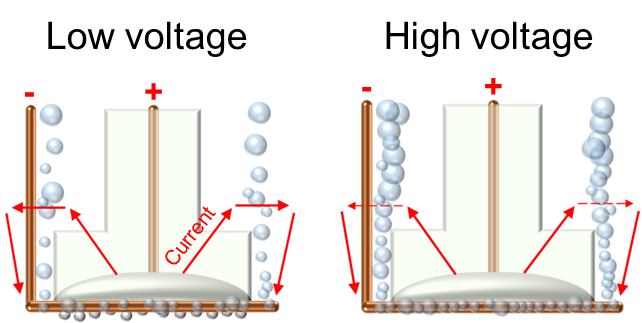


**Figure S8.** The denser bubble wall generated by high voltage causes smaller current in device.


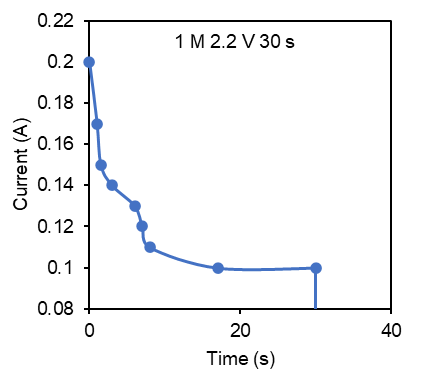


**Figure S9.** Current-time curve of the power supply under applied oxidizing voltage of 2.2 V for 30 s.

**
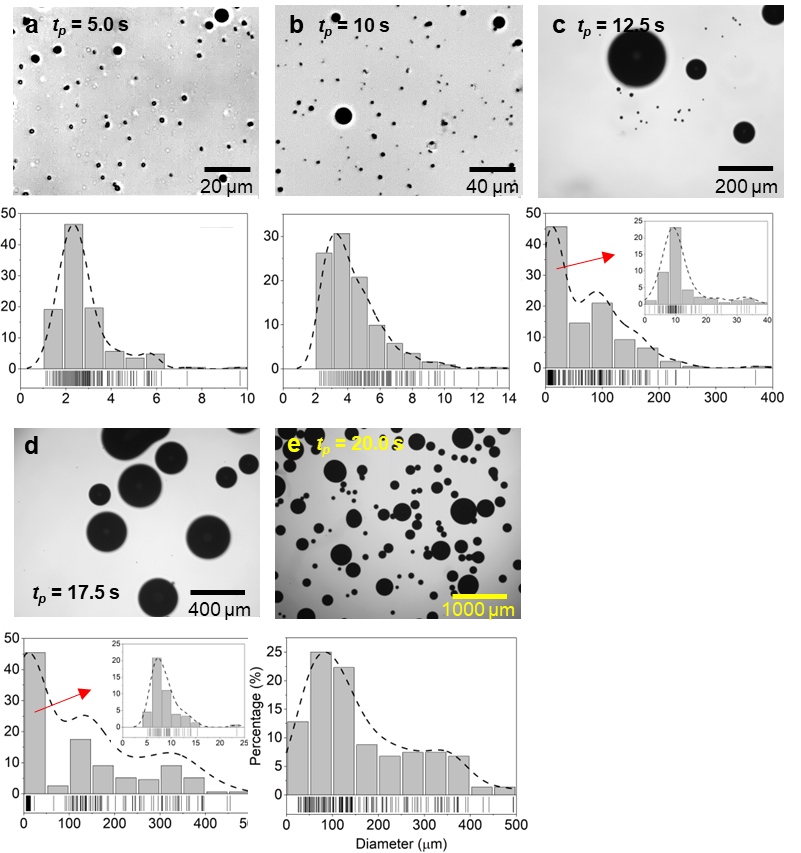
**

**Figure S10.** SEM images and size distribution of the LMMPs using the LM sieve under *t_p_* = (A) 5 s, (B) 10 s, (C) 12.5 s, (D) 17.5 s, and (E) 20 s.

**
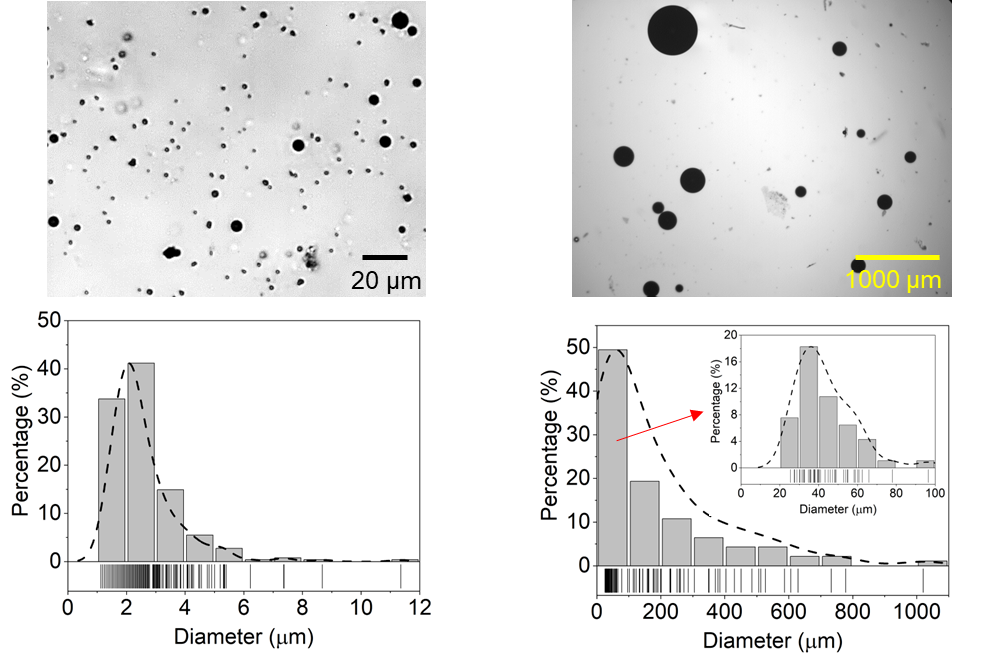
**

**Figure S11.** The size distribution and SEM image of EGaIn LMMPs producing by 25 µm nylon sieve when *t_p_* = 2.5 s (left) and 11 s (right).

**
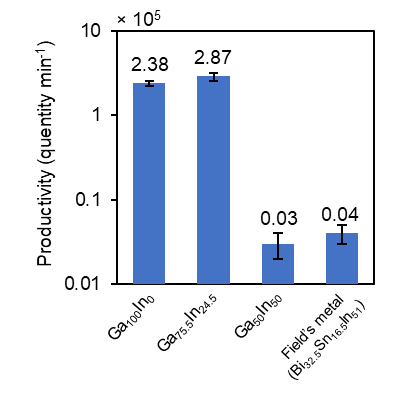
**

**Figure S12.** Productivity of different LMMPs with hole size of 25 µm_。_


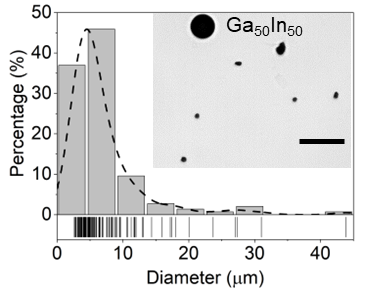


**Figure S13.** Size distribution and SEM images of Ga_50_In_50_.


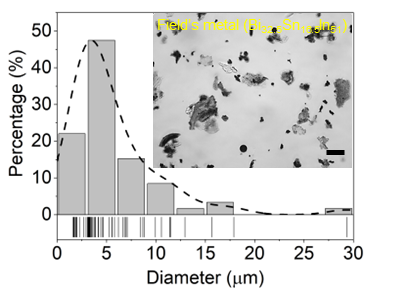


**Figure S14.** Size distribution and SEM images of Field’s metal (Bi_32.5_Sn_16.5_In_51_).

**
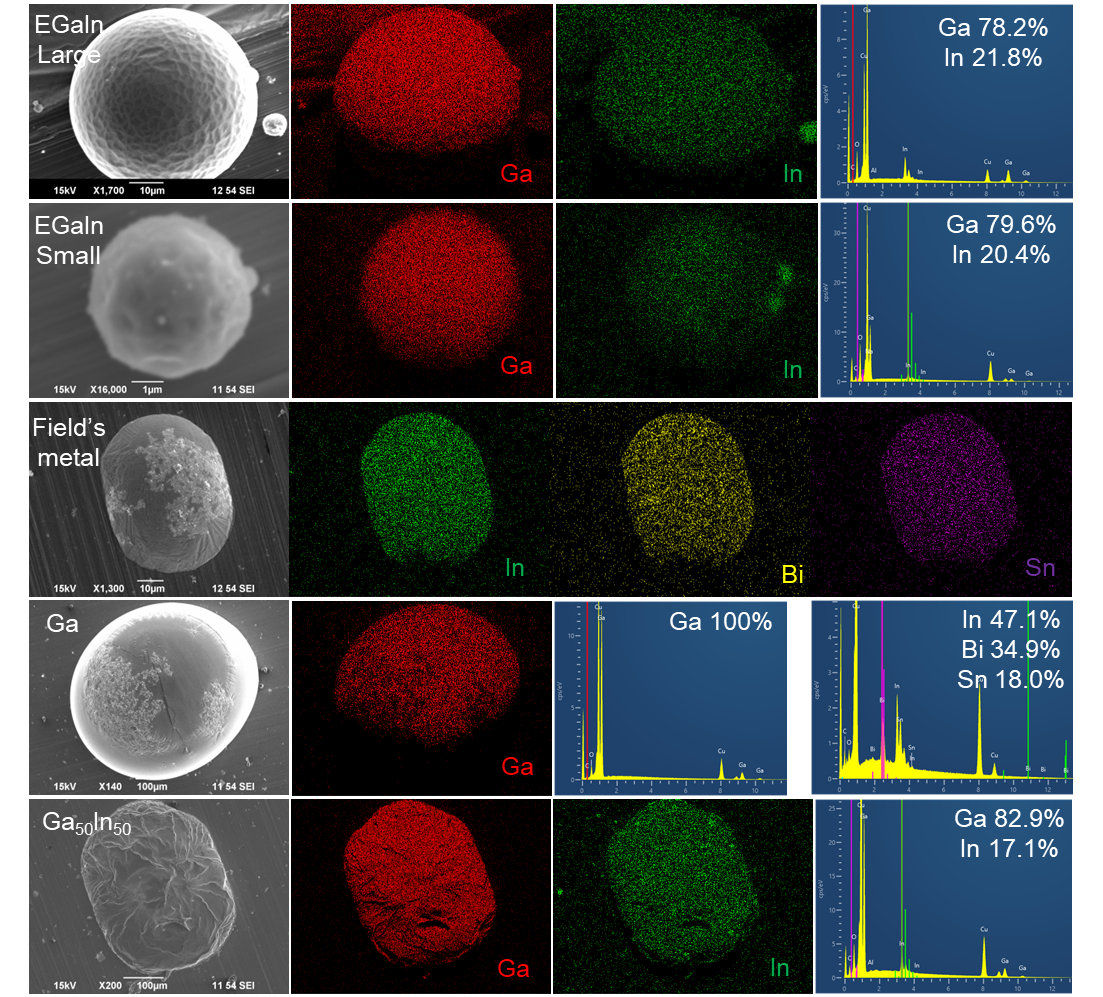
**

**Figure S15.** The EDS and component proportions of different LMMPs.

**
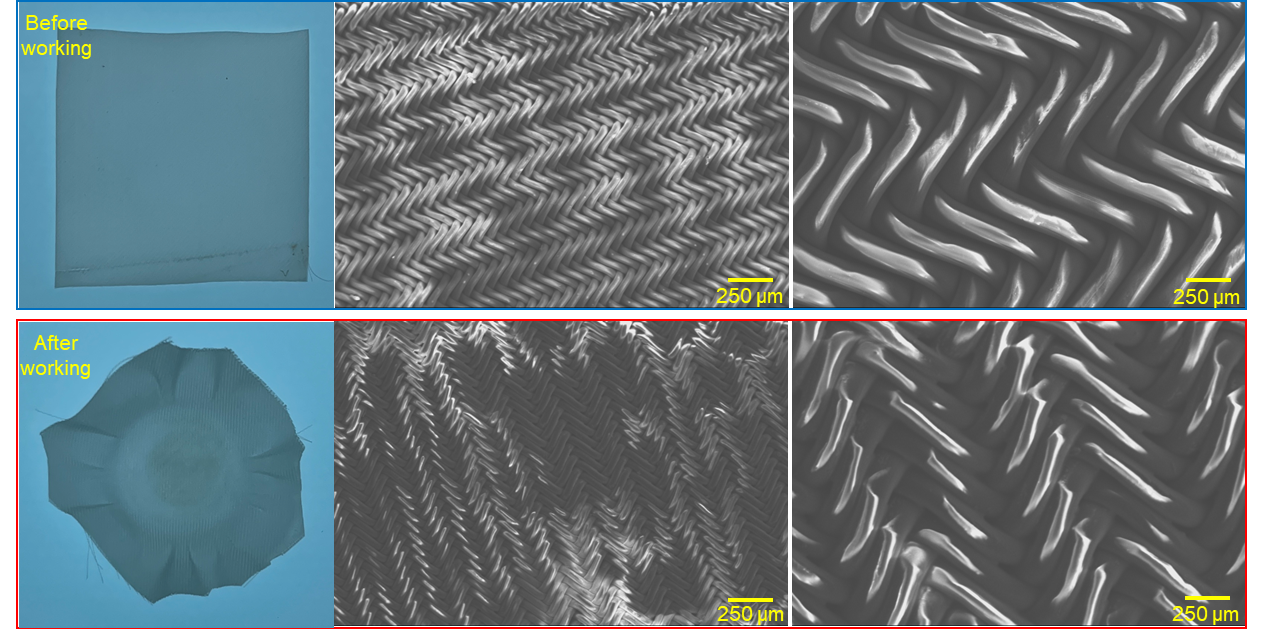
**

**Figure S16.** Optical and SEM images of filter sieve before and after LMMPs generating. The filter sieve after working is treated by a NaOH cleaning process.

**
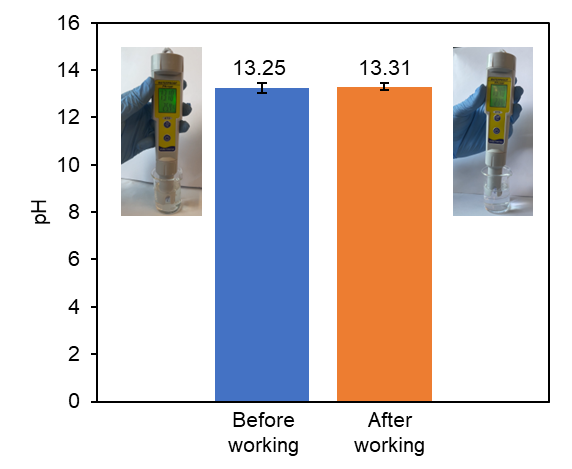
**

**Figure S17.** pH values of NaOH solution before ana after working.

**
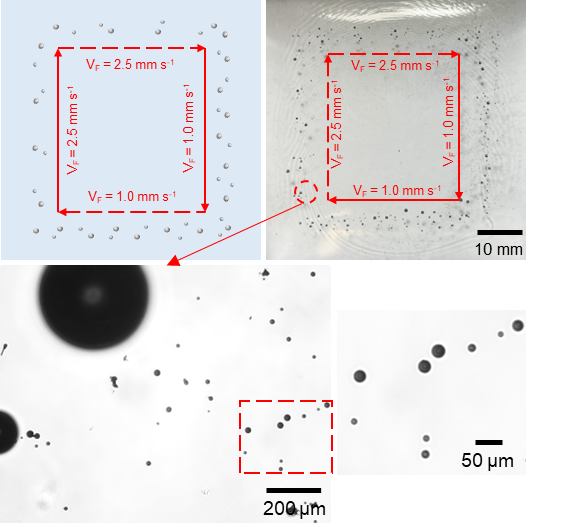
**

**Figure S18.** Square pattern of LM droplets under customized design with same oxidizing power time (*t_p_* = 2.5 s) but changed V_F_.


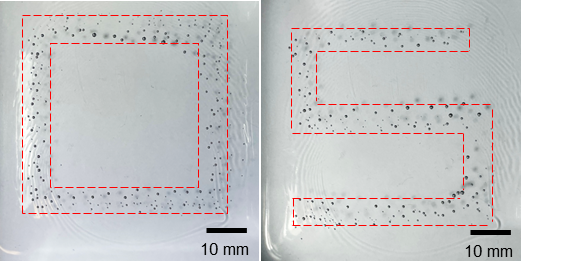


**Figure S19.** Programmable patterned LM droplets with the different shapes of square (left) and “S” (right).

**
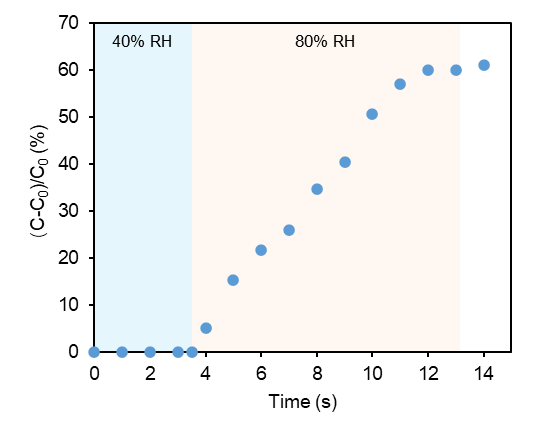
**

**Figure S20.** Relative capacitance change-time curve of LMMP-based humidity sensor. The response time is 8 s.

**
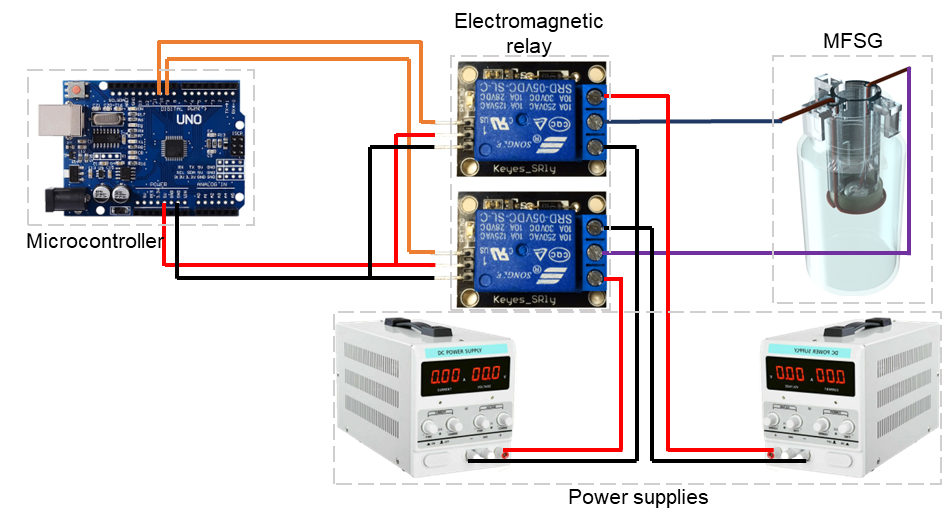
**

**Figure S21.** Schematic diagram of the installation including power supply and control system of the device.

**Table S1.** Comparison of fabrication methods for fabricating LMMPs.

| **Device** | **Mechanism** | **Simplicity** | **Size range/distribution** | **Monodispersity** | **Productivity** | **Energy conservation** | **Ref.** |
| --- | --- | --- | --- | --- | --- | --- | --- |
| Ultrasonic probe | Acoustics | *** | 20–1000 nm  (After centrifuging) | *  (After centrifuging) | ***** | *  (300 W for 20 min) | [10a] |
| Piezoelectric transducer | Acoustics | *** | 80-500 nm  (After centrifuging) | **  (After centrifuging) | ***** | **  (11 W for 20 min) | [10b] |
| Stirring stick | Shearing | ***** | 8-40 µm | ** | **  (~30 particles s^-1^) | ***  (18 W for 5 min) | [13e] |
| Revolving needle emulsion generator | Shearing | **** | 270-450 µm | **** | **  (~18 particle s^-1^) | ***  (30 W) | [13a] |
| Spinning conical frustum | Shearing | **** | 200-320 µm | **** | ***  (~230 particle s^-1^) | ***  (30 W) | [13c] |
| Microfluidic chip | Flow focusing | * | ~105 µm | ***** | ***  (~458 particles s^-1^) | ***** | [9] |
| Electrical controlled microfluidic chip | Flow focusing and electrohydrodynamic | * | 80-186 µm | ***** | ***  (~215 particles s^-1^) | ***  (0-18 V) | [8b] |
| Laser-cut PDMS mold | Molding | ** | 100–3500 μm | *** | * | *** | [17] |
| Micro-syringe pump and electric field | Electrohydrodynamic | *** | 380-600 μm/  546.18 ± 13.11 μm | *** | **  (~10 particles s^-1^) | *****  (0.65 mA) | [14b] |
| MFSG | Electrohydrodynamic | ***** | 2.39-320.07 μm /2.39 ± 0.90 μm | **** | *****  (~2170 particles s^-1^) | *****  (0.21 W for 5 min) | This work |

^1^ The number of asterisks (*) represents degree of each characteristic; 1 means relatively low, while 5 means high.

**Table S2.** The size and polydispersity of LMMPs with different *t_p_*.

| *t_p_* (s) | Diameter (µm) | PDI* |
| --- | --- | --- |
| 1 | 2.56 ± 0.82 | 0.10 |
| 2.5 | 2.39 ± 0.90 | 0.14 |
| 5 | 2.59 ± 1.18 | 0.21 |
| 10 | 4.33 ± 1.78 | 0.17 |
| 12.5 | First peak:  11.90 ± 7.23  Second peak:  113.90 ± 53.30 | First peak:  0.37  Second peak:  0.22 |
| 15 | First peak:  8.91 ± 3.62  Second peak:  174.52 ± 64.52 | First peak:  0.16  Second peak:  0.14 |
| 17.5 | First peak:  8.67 ± 2.94  Second peak:  148.56 ± 42.70  Third peak:  335.66 ± 51.52 | First peak:  0.12  Second peak:  0.08  Third peak:  0.02 |
| 20 | First peak:  96.31 ± 45.99  Second peak:  312.64 ± 73.25 | First peak:  0.23  Second peak:  0.05 |
| 30 | First peak:  91.59 ± 38.1  Second peak:  320.07 ± 84.48 | First peak:  0.17  Second peak:  0.07 |

*PDI: Polydispersity Index

$$PDI= \frac{{Standard deviation}^{2}}{{Mean}^{2}}$$

**Table S3.** Comparison of flexible humidity sensors.

| Sensing materials | Sensing mechanism | Sensitivity | Response time | Fabrication simplicity | Ref. |
| --- | --- | --- | --- | --- | --- |
| PEDOT:PSS nanowires | Resistive | 0.48/%RH | 0.63 s | PDMS Mold Nanoimprinting with Capillary Nanowire Deposition  * | [31d] |
| SnO_2_/MoS_2_ | Capacitance | 352pF/%RH | 13 s | Two-step hydrothermal route  * | [31b] |
| Reduced graphene oxide/WS_2_ | Resistive | 0.18/%RH | 35 s | Spray method and thermal reduction treatment  ** | [31c] |
| Multi-walled CNT | Impedance | 1.1/%RH | ~200 s | Evaporative casting  **** | [31a] |
| LMMPs | Capacitance | GF = 1.5  (1.5/%RH) | 8 s | Electrochemistry and automatic platform  **** | This work |

^1^ The number of asterisks (*) represents a degree of simplicity.

[17] M. R. Khan, C. B. Eaker, E. F. Bowden, M. D. Dickey, *Proc Natl Acad Sci U S A* **2014**, *111*, 14047.

[31] a) X. Peng, J. Chu, A. Aldalbahi, M. Rivera, L. Wang, S. Duan, P. Feng, Applied Surface Science 2016, 387, 149; b) Y. Zhao, B. Yang, J. Liu, Sensors and Actuators B: Chemical 2018, 271, 256; c) Z.-H. Duan, Q.-N. Zhao, C.-Z. Li, S. Wang, Y.-D. Jiang, Y.-J. Zhang, B.-H. Liu, H.-L. Tai, Rare Metals 2020, 40, 1762; d) C. Zhou, X. Zhang, N. Tang, Y. Fang, H. Zhang, X. Duan, Nanotechnology 2020, 31, 125302.
